# Exacerbation by knocking-out metallothionein gene of obesity-induced cardiac remodeling is associated with the activation of CARD9 signaling

**DOI:** 10.7150/ijbs.105513

**Published:** 2025-01-13

**Authors:** Haina Zhang, Wenqian Zhou, Xiang Wang, Hongbo Men, Jiqun Wang, Jianxiang Xu, Shanshan Zhou, Quan Liu, Lu Cai

**Affiliations:** 1 Pediatric Research Institute, Department of Pediatrics, University of Louisville, Louisville, KY, 40202, USA.; 2 Department of Cardiology, The Second Hospital of Jilin University, Jilin University, Changchun, 130041, China.; 3 Department of Cardiovascular Diseases, The First Hospital of Jilin University, Jilin University, Changchun, 130021, China.; 4 Departments of Radiation Oncology, Pharmacology and Toxicology, University of Louisville, Louisville, KY, 40202, USA.

**Keywords:** Obesity cardiomyopathy, Cardiac remodeling, Metallothionein, CARD9, Inflammation

## Abstract

Obesity increases the risk of metabolic syndrome including insulin resistance, dyslipidemia, and cardiovascular disease. We demonstrated insulin resistance, cardiac hypertrophy, and cardiac inflammation in an obese mouse model induced by a high-fat diet (HFD). Caspase recruitment domain-containing protein 9 (CARD9) and B-cell lymphoma/leukemia 10 (BCL10) were upregulated, and p38 MAPK was activated in these mice. Zinc supplementation prevented these changes with upregulation of metallothionein (MT). Deletion of *MT* exacerbated palmitate-triggered expression of BCL10 and p38 MAPK activation and eliminated the protective benefits of zinc in palmitate-treated cardiomyocytes. Here we further investigated the mechanisms by which endogenous MT expression affects HFD-induced cardiac remodeling and the CARD9/BCL10/p38 MAPK pathway. Male *MT* knockout and 129S wild-type mice were assigned to receive either a normal diet or a HFD from 8-week-age for 18 weeks. *MT* knockout (KO) aggravated HFD-induced obesity and systemic metabolic disorder, reflected by increased body weight, perirenal white adipose tissue, and plasma cholesterol, and cardiac hypertrophy and fibrosis. Obese *MT*-KO mice had abundant cardiac macrophages, upregulated cardiac proinflammatory cytokines, chemokines, adhesion molecules, CARD9, and BCL10 and activated NF-κB. *MT*-KO exacerbated HFD-induced trace metal dyshomeostasis and oxidative stress. *MT*-KO combined with HFD-induced obesity synergistically promotes cardiac remodeling, possibly via trace metal dyshomeostasis-induced oxidative stress to trigger CARD9/BCL10-mediated NF-κB activation.

## Introduction

Obesity is the most common chronic medical condition worldwide. It affects over 1 billion people globally, based on the latest published data 1. Metabolically abnormal obesity (MAO) is a disorder affecting blood pressure, glucose, and lipid metabolism. This disorder contributes to various cardiovascular diseases (CVDs) such as coronary artery disease (CAD), hypertension, and cardiac dysfunction or even heart failure (HF), called obesity cardiomyopathy 2. Obesity cardiomyopathy is characterized by cardiac metabolic, structural, and functional abnormalities mediated by obesity alone without other CVDs such as hypertension and CAD 3. These abnormalities include cardiac pathological remodeling and impaired systolic and diastolic function. These dysfunctions then develop into HF, including those with preserved ejection fraction and HF with reduced ejection fraction 3,4. Obesity cardiomyopathy is becoming a major global public health problem without efficacious therapeutic targets. It's a progressive process from metabolically healthy but obese to MAO and cardiac remodeling. Hence, early identification and prevention of MAO are vital for the prevention of obesity cardiomyopathy.

Obesity cardiomyopathy is associated with systemic and local metabolic disturbances, inflammation, and subcellular abnormalities such as oxidative stress, endoplasmic reticulum stress, mitochondrial dysfunction and autophagy 3,5. Thus, local and systemic chronic inflammation may have pivotal roles in the occurrence and progression of obesity cardiomyopathy 5.

Expressed on myeloid cells, caspase recruitment domain-containing protein 9 (CARD9) contributes to the transduction of signals related to innate immunity and the inflammatory response, thereby protecting against pathogen invasion 6. When activated, CARD9 forms the signaling complex CARD9/B-cell lymphoma/leukemia 10 (BCL10)/mucosa-associated lymphoid tissue 1 (MALT1) (CBM) with BCL10 and MALT1. Then MAPKs and/or NF-κB were activated to mediate proinflammatory cytokine and chemokine production 7. CARD9 promotes inflammation in CVDs, including HF, myocardial infarction, viral myocarditis, cardiac arteritis, myocardial ischemia/reperfusion, hypertension, and obesity cardiomyopathy 6,8,9. We previously reported the regulation of CARD9 and BCL10 expression in the hearts of mice with obesity and diabetic cardiomyopathy. In both cases, cytokine production and p38 MAPK activation were augmented 10,11. Furthermore, CARD9 deletion in mice with metabolic disorders and obesity improved glucose intolerance, decreased cardiac fibrosis and cardiac dysfunction, reduced cardiac macrophage infiltration into the heart, lowered proinflammatory cytokine production, and attenuated MAPK activity 6,12,13.

Metallothionein (MT) is a protein characterized by a high cysteine content and ability to bind metals. Its roles include zinc (Zn), copper (Cu), and other metal homeostasis maintaining, free radical scavenging, oxidative stress and damage protecting, and heavy metal detoxifying 14. Polymorphisms in *MT1A* gene have been linked to a heightened susceptibility to type 2 diabetes mellitus. Meanwhile, diabetics have higher levels of proinflammatory cytokines than healthy participants 15. Moreover, we demonstrated that MT upregulation by Zn supplementation and cardiac-specific *MT* overexpression prevented diabetic cardiomyopathy (DCM) 16,17. Conversely, *MT* deletion aggravated diabetes-induced oxidative damage, inflammation, and cardiac remodeling 18.

Owing to the common link of MT and CARD9 signaling with inflammation, we preliminarily explored these interactions in obesity cardiomyopathy. We observed that obesity induces cardiac hypertrophy by activating p38 MAPK through CARD9/BCL10 complex upregulation, which was assumed to be mediated by increased oxidative stress 10,19. *In vitro*, MT activation by Zn supplementation ameliorated palmitate-induced oxidative stress, upregulated BCL10 expression, and p38 MAPK activation in wild type (WT) cardiomyocytes. However, *MT* deletion counteracted the beneficial effect of Zn on palmitate-induced BCL10 overexpression and p38 MAPK activation 10,19.

Here, we used *MT* knockout (KO) mice to explore whether MT protects against HFD-induced obesity cardiomyopathy via the CARD9/MAPKs pathway *in vivo*. We further investigated the mechanisms through which this protein activates CARD9 signaling. As NF-κB is a downstream component of CARD9 that is in parallel with p38 MAPK, we examined whether its activation is implicated in obesity cardiomyopathy.

## Materials and Methods

### Animals and experimental models

*MT-*KO mice were generated by interbreeding homozygous mutants (*MT*-/-, both *MT1* and *MT2* gene knockout) within a 129S1 WT background. All breeding pairs (Jackson Laboratory, Bar Harbor, ME, USA), were mated according to the vendor's instructions. Male WT 129S1 (*MT*+/+) mice were also procured from Jackson Laboratory. All experimental procedures were approved by the Institutional Animal Care and Use Committee of the University of Louisville (Louisville, KY, USA) which is certified by the American Association for the Accreditation of Laboratory Animal Care.

These mice were started on a normal diet (ND; 10% kcal from fat; No. D14020202, Research Diets, New Brunswick, NJ, USA) or a high-fat diet (HFD; 60% kcal from fat; No. D14020205, Research Diets) at 8 weeks and maintained on each diet for 18 weeks. Weekly body weight (BW) measurements were taken. The mice were anesthetized through an intraperitoneal avertin injection (250 mg/kg) after an 18-week dietary intervention, and blood samples were obtained from the inferior vena cava. The mice were then euthanized by exsanguination. Heart weight, tibia length, and peri-renal white adipose tissue weight were recorded, and the tissues were harvested.

### Intraperitoneal glucose tolerance test (IPGTT)

The IPGTT was conducted at 26 weeks of age. Mice were subjected to a 6-h fast that began at 8 a.m. and ended at 2 p.m., followed by an intraperitoneal administration of glucose solution (2 g/kg BW). Blood glucose levels were measured at 0, 15, 30, 60, 90, and 120 minutes post-glucose administration, with a FreeStyle Lite glucometer (Abbott Diabetes Care, Alameda, CA, USA).

### Echocardiography

At the end of the study, transthoracic echocardiography was conducted utilizing a high-resolution imaging system (Vevo 770; Visual Sonics, Toronto, ON, Canada) as previously described 20. Left ventricular (LV) parameters, including LV internal dimension (LVID), LV posterior wall thickness (LVPW), and interventricular septum (IVS), were measured. LV mass and volume, ejection fraction (EF), and fractional shortening (FS) were calculated using Vevo 770 software.

### Assessment of metal concentration in the heart tissue

Zn, iron (Fe), and Cu concentrations in the heart tissue were determined by inductively coupled plasma mass spectrometry (ICP-MS; X series II, Thermo Fisher Scientific, Waltham, MA, USA). Each sample underwent digestion with 0.6 mL of concentrated nitric acid (70%) under conditions of 85 °C for a duration of 4 hours. After cooling to room temperature, the samples experienced 1 minute of centrifugation at 5,000 rpm and dilution with 9 mL deionized Milli-Q water (EMD Millipore, Billerica, MA, USA) (constituting a 4.38% nitric acid solution), which was then vortexed and subjected to ICP-MS analysis.

### Biochemical plasma analysis

Plasma triglyceride and cholesterol levels were measured utilizing Infinity™ triglyceride/cholesterol liquid stable reagents (Cat#TR22421, Cat#TR13421; Thermo Fisher Scientific), following the provided protocols. Briefly, the plasma samples underwent a 1:100 dilution using the aforementioned reagents, followed by thorough mixing and incubation at 37 ℃ for 5 minutes. Subsequently, a microplate reader measured the absorbance at 500 nm.

### Histopathological analysis

The heart tissue was embedded in paraffin and sectioned as previously described 10. The tissues were fixed in 10% buffered formalin, followed by paraffin embedding and sectioning at a thickness of 5 μm. To determine the cross-sectional areas of the cardiomyocytes and collagen deposition in cardiac tissues, we used fluorescein isothiocyanate (FITC)-conjugated wheat germ agglutinin (WGA; Alexa Fluor 488 conjugate; Molecular Probes/Invitrogen, Carlsbad, CA, USA) and Picro-Sirius Red staining. Immunohistochemical (IHC) staining with anti-CD68 (1:150 dilution; Abcam, Cambridge, MA, USA) was performed to examine cardiac macrophage infiltration. All stained sections were examined with an Olympus BX43 microscope (Olympus Life Science, Tokyo, Japan), and quantitative measurements were performed using ImageJ software.

### Western blot analysis

Western blotting was performed as previously described 10. Heart tissues were collected and lysed. The protein samples were separated by SDS-PAGE and then electrotransferred onto nitrocellulose membranes. The membranes were incubated with primary antibodies, followed by appropriate secondary antibodies (Santa Cruz Biotechnology, Dallas, TX, USA). Images were acquired using a ChemiDoc Touch Imaging System (Bio-Rad Laboratories). Protein content was determined with Image Lab software (Bio-Rad Laboratories) and normalized against the respective controls. A modified western blotting protocol, as described before, was employed for the evaluation of MT expression 16.

Primary antibodies used included those against TGF-β1 (1:1000; ab92486; Abcam, Cambridge, MA, USA), CD68 (1:1000; ab125212), vascular cell adhesion molecule-1 (VCAM-1; 1: 1000; ab134047), intercellular adhesion molecule-1 (ICAM-1; 1:1000; ab179707), IL-6 (1:1000; ab208113), TNF-α (1:1000; ab6671), IL-1β (1:1000; ab9722), GAPDH (1:3000; ab37168), BCL10 (1 : 1000; sc-5611; Santa Cruz Biotechnology), collagen1A1 (COL1A1; 1:500; SC293182), CARD9 (1:1000; 12283s; Cell Signaling Technology(CST), Danvers, MA, USA), phosphorylation-NF-κB p65 (p-p65; 1:1000; 3033s; CST), p-p38 MAPK/p38 MAPK (p-p38/p38; 1:1000; 9211s/9212s; CST), p-JNK/JNK (1:1000; 9255s/9252s; CST), p-Erk1/2/Erk1/2 (1:1000; 4370s/4696s; CST), 3-NT (1:1000; ab5411; EMD Millipore, Billerica, MA, USA), 4-HNE (1:2000; Alpha Diagnostic International, San Antonio, TX, USA), MT (1:1000; M0639; Dako, Glostrup, Denmark).

### Quantitative real-time polymerase chain reaction (qRT-PCR)

TRIzol reagent (Invitrogen) was used to extract total RNA from heart tissues, followed by assessment of RNA purity and concentration using a NanoDrop ND-1000 spectrophotometer. A reverse transcription kit (Promega, Madison, WI, USA) was then used to synthesize cDNA from 1 μg total RNA, following the provided instructions. The qRT-PCR was performed in a 10 μl solution including TagMan universal PCR master mix (Invitrogen) using a LightCycler 96 RT-PCR system (Roche Diagnostics, Indianapolis, IN, USA). Primers for GAPDH (Mm99999915_g1), collagen I (col1a1, Mm00801666_g), collagen III (col3a1, Mm00802300_m1), fibronectin, (Fn, Mm01256744_m1), TGF-β (Mm01178820_m1), monocyte chemoattractant protein-1 (MCP-1/CCL2, Mm00441242_m1), and CD68 (4331182 Mm03047343_m1) were acquired from Thermo Fisher Scientific.

### Statistical analysis

The data were processed using GraphPad Prism v. 8 (San Diego, CA, USA). Data are presented as mean ± standard deviation (SD; n = 5). Between-group differences were determined by one-way analysis of variance (ANOVA) followed by Tukey's post-hoc test or two-way ANOVA. P < 0.05 indicated statistically significant difference.

## Results

### *MT* knockout exacerbated HFD-induced obesity and metabolic disorders

**Figure 1:**
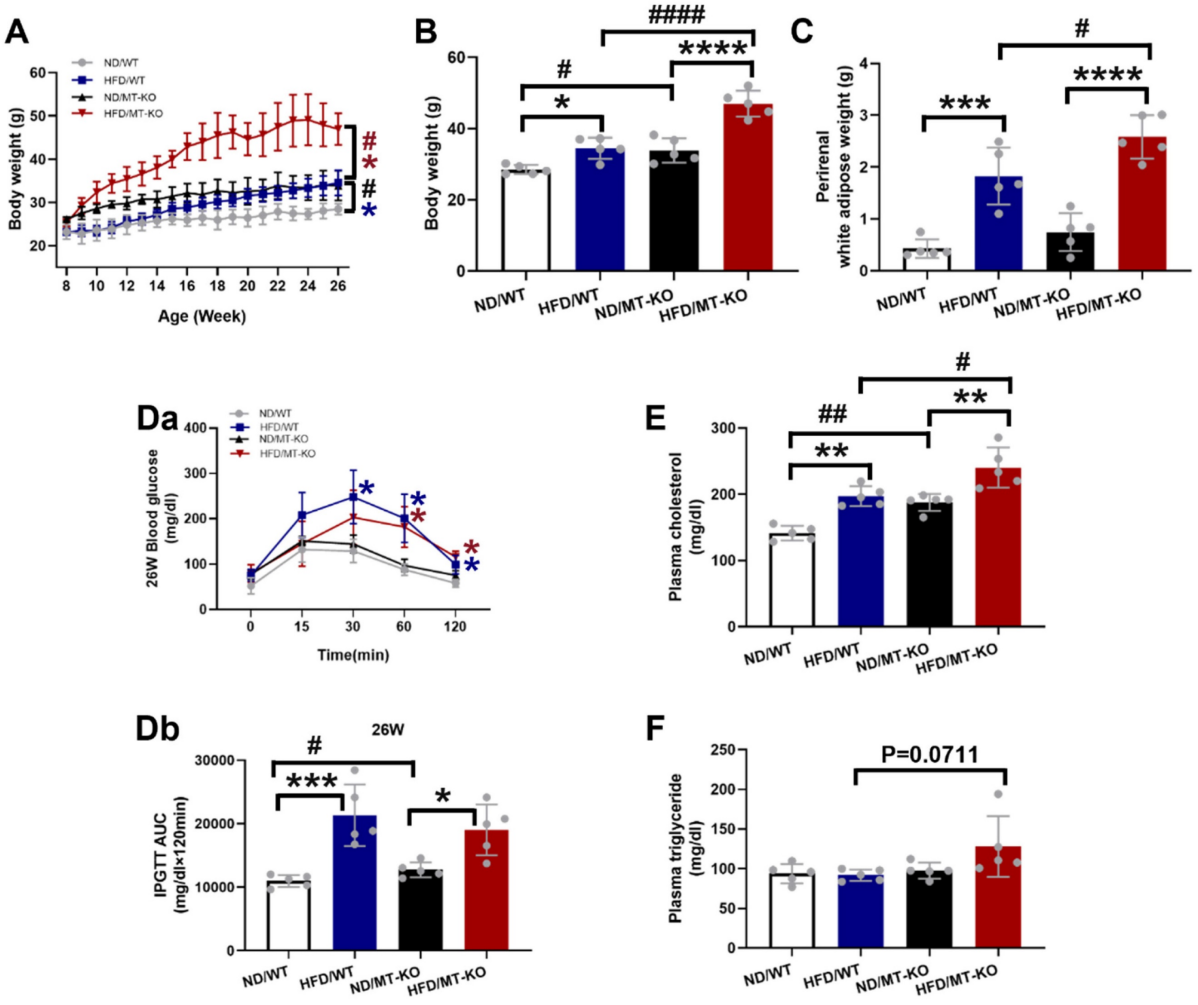
**The effect of *MT* knockout on HFD-induced obesity and metabolic disorders.** A: Body weights during 26 weeks of ages and 18 weeks of HFD feeding (started at 8 weeks old). B-C: Weight of body and perirenal adipose tissue weight at the end of 18-week feeding. D: The changes of blood glucose levels over time after IPGTT in 26-week-old mice (Da, i.e., at the end of 18-wks HFD feeding) and the IPGTT results via calculation of the integrated area under the curves (AUC, Db). E-F: Plasma cholesterol and triglyceride. Data are presented as Mean ± SD (n=5). *P<0.05, **P<0.01, ***P<0.001, ****P<0.0001, HFD vs. ND in WT and *MT*-KO groups; ^#^P<0.05,^ ##^P<0.01,^ ####^P<0.0001, *MT*-KO vs. WT in ND and HFD groups.

Fig. 1A-C shows that both WT and *MT*-KO mice fed a HFD for 18 weeks displayed a significant increase in BW and perirenal white adipose tissue weight in comparison to their corresponding ND-fed mice. Moreover, HFD-fed caused significantly different results between WT and *MT*-KO mice. In addition, *MT*-KO alone resulted in a significantly elevated BW relative to WT mice fed a ND (Fig. 1A, B). These outcomes propose that *MT* deletion increased BW and perirenal white adipose accumulation in response to HFD feeding, i.e., aggravated HFD-induced obesity.

As shown in Fig. 1D, *MT*-KO or obese mice showed significantly reduced glucose tolerance, as reflected by a larger area under the curve at 26 weeks of age (i.e. ,18 weeks of HFD feeding) in both WT and *MT*-KO strains. Combined *MT*-KO and HFD did not significantly worse glucose tolerance compared to either HFD/WT or *MT*-KO alone group (P > 0.05).

The obese mice displayed augmented plasma cholesterol in the WT and *MT*-KO groups compared to their corresponding ND-fed mice. Moreover, the *MT-*KO obese mice exhibited higher plasma cholesterol levels than the WT mice fed with HFD (Fig. 1E). As for plasma triglyceride, neither *MT* deletion nor HFD has effect, but obese *MT*-KO mice showed slightly increased plasma triglyceride levels compared to other groups (Fig. 1F).

### *MT* gene knockout promoted HFD-induced cardiac remodeling

Echocardiographic analysis revealed no significant impact on cardiac dysfunction after 18 weeks of exposure to a HFD, as indicated by functional parameters EF% and FS%, in WT and *MT-*KO mice (Table 1). As cardiac structural parameters, IVS, LV mass, and LVPW (Table 1) were significantly increased in HFD/*MT-*KO group than in HFD/WT and ND/*MT-*KO groups. This indicated that *MT* gene KO combined with HFD-induced obesity caused cardiac structure remodeling.

**Figure 2:**
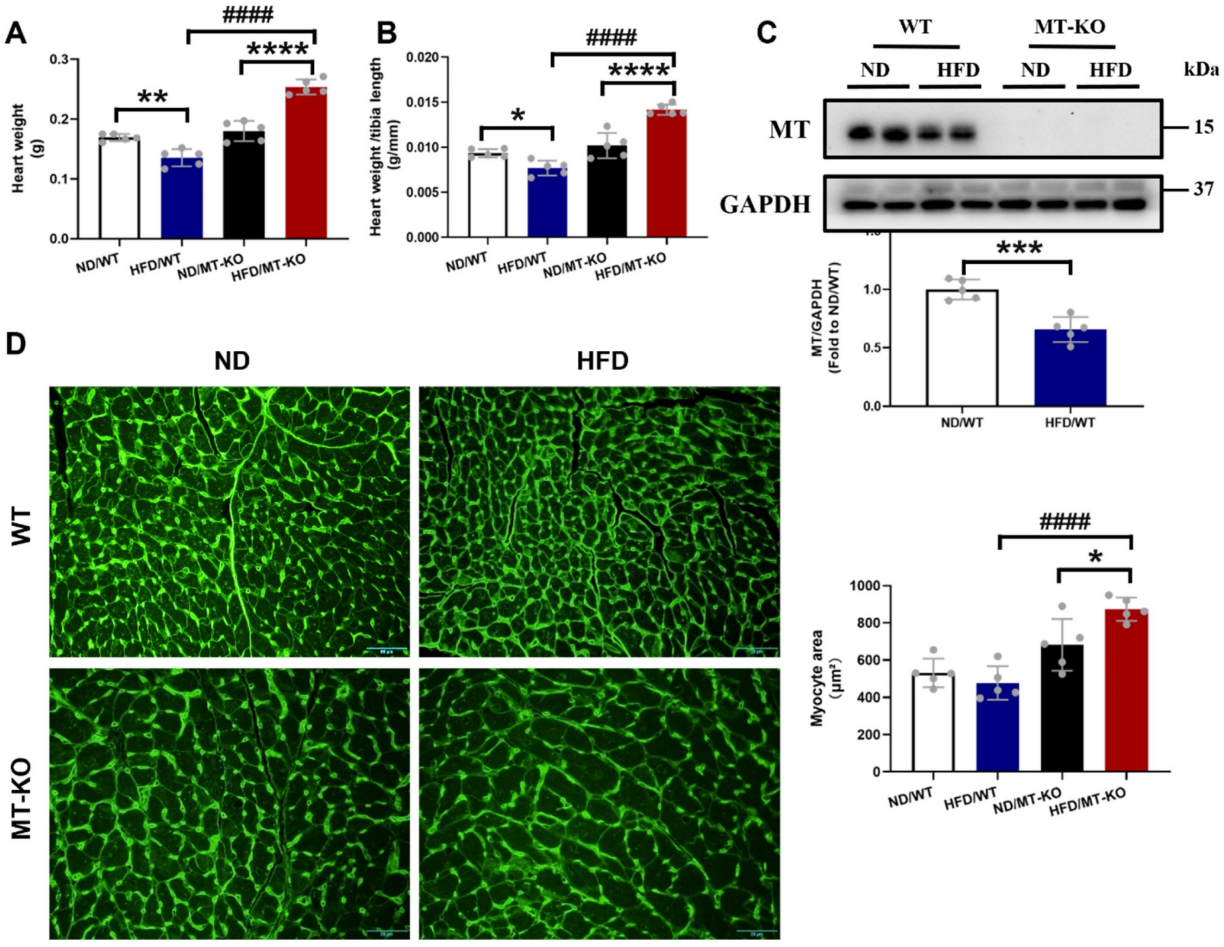
** The effect of *MT* knockout on HFD-induced cardiac hypertrophy.** A-B, Heart weight and heart weight to tibia length ratio. C, Cardiac MT protein expression evaluated by Western blot. D, WGA staining of cardiac tissue sections and the quantification of myocyte cross-sectional areas (scale bar = 50 μm). Data are presented as Mean ± SD (n=5). *P<0.05, **P<0.01, ***P<0.001, ****P<0.0001, HFD vs. ND in WT and *MT*-KO groups, ^####^P<0.0001, *MT*-KO vs. WT.

Consistent with cardiac hypertrophy, estimated by echocardiographic LV mass, *MT-*KO obese mice showed significantly higher heart weight (HW, Fig. 2A) and HW/tibia length ratio (Fig. 2B) than those in two corresponding control groups. We confirmed the absence of the cardiac MT abundance in *MT-*KO mice with Western blotting analysis since MT expression was undetectable in the hearts of *MT*-KO mice, whereas it was strongly evident in the WT mice (Fig. 2C). Figure 2C also shows that HFD exposure for 18 weeks significantly downregulated MT in the WT mice. Finally, we used WGA staining to confirm that the cardiomyocytes in HFD/*MT-*KO group were enlarged compared to those in the other two control groups (Fig. 2D).

**Figure 3:**
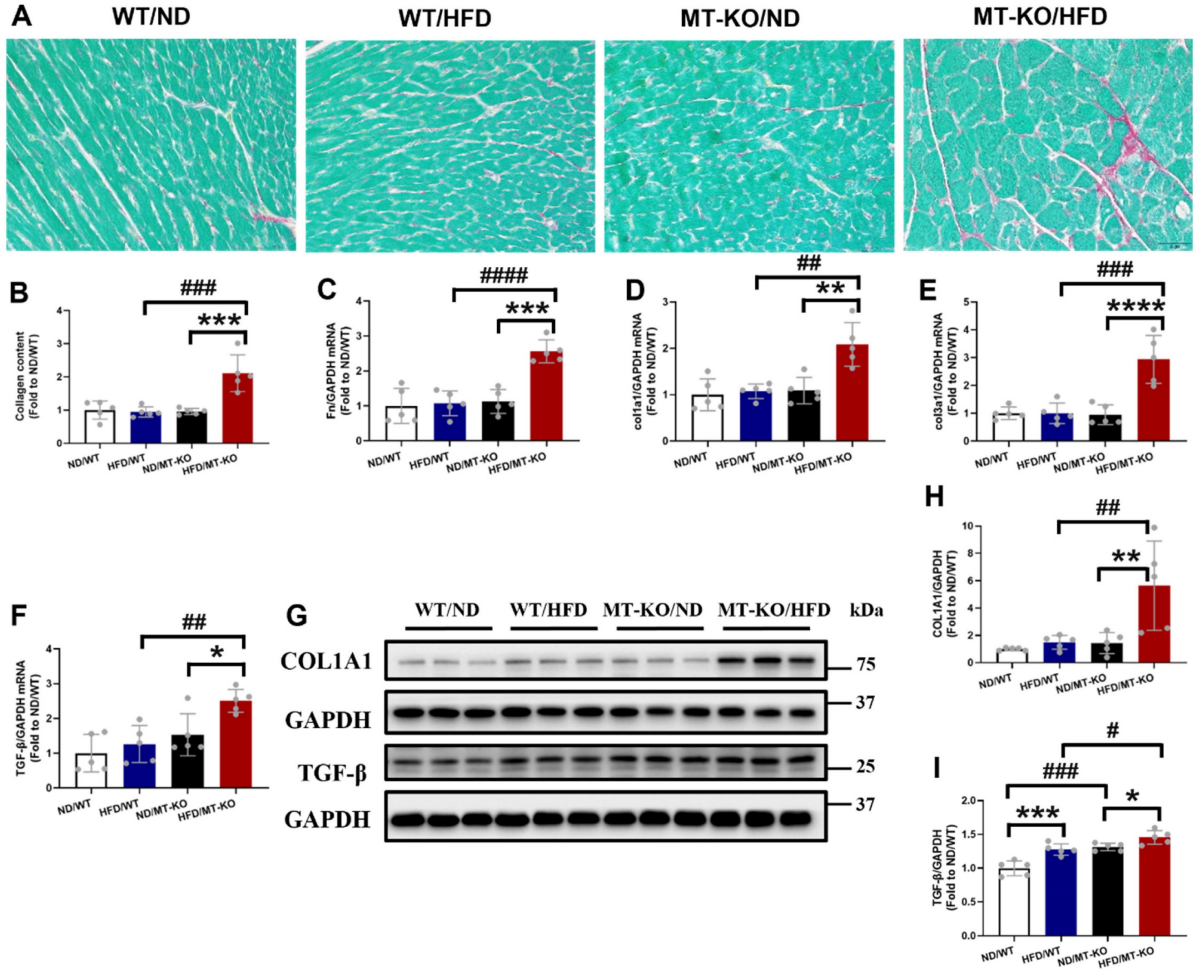
** The effect of *MT* knockout on HFD-induced cardiac fibrosis.** A, Picro-Sirius Red staining results of heart tissue sections. B, Quantitative analysis of Picro-Sirius Red staining for collagen accumulation. C-F: Cardiac pro-fibrotic mRNA expression of Fn, col1a1, col3a1, and TGF-β evaluated by qRT-PCR. G-I, Protein expression of COL1A1 and TGF-β1 detected by Western blot. Data are presented as Mean ± SD (n=5). *P<0.05, **P<0.01, ***P<0.001, ****P<0.0001, HFD vs. ND in WT and *MT*-KO groups, ^#^P<0.05,^ ##^P<0.01,^ ###^P<0.001,^ ####^P<0.0001, *MT*-KO vs. WT in ND and HFD groups.

Picro-Sirius Red staining, a general fibrotic index, showed increased collagen accumulation in the hearts of HFD/*MT-*KO group compared to HFD/WT and ND/*MT-*KO groups (Fig. 3A, B). Meanwhile, the *MT*-KO obese group exhibited markedly elevated mRNA expression levels of *FN*, *Col1a1*, *Col3a1*, and *TGF-β* (Fig. 3C-F) compared to the HFD-fed WT and ND-fed *MT-*KO groups. Furthermore, the protein abundance of the profibrotic mediators collagen1A1 (Fig 3G, H) and TGF-β1 (Fig. 3G, I) was consistent with their mRNA expression levels (Fig.3 E, F). Thus, *MT*-KO promoted cardiac hypertrophy and fibrosis in HFD-induced obesity in the mouse model.

### Potential mechanisms by which *MT* knockout exacerbated HFD-induced obesity cardiomyopathy

#### *MT* gene knockout increased HFD-induced macrophage infiltration and inflammation in mouse cardiac tissue

**Figure 4:**
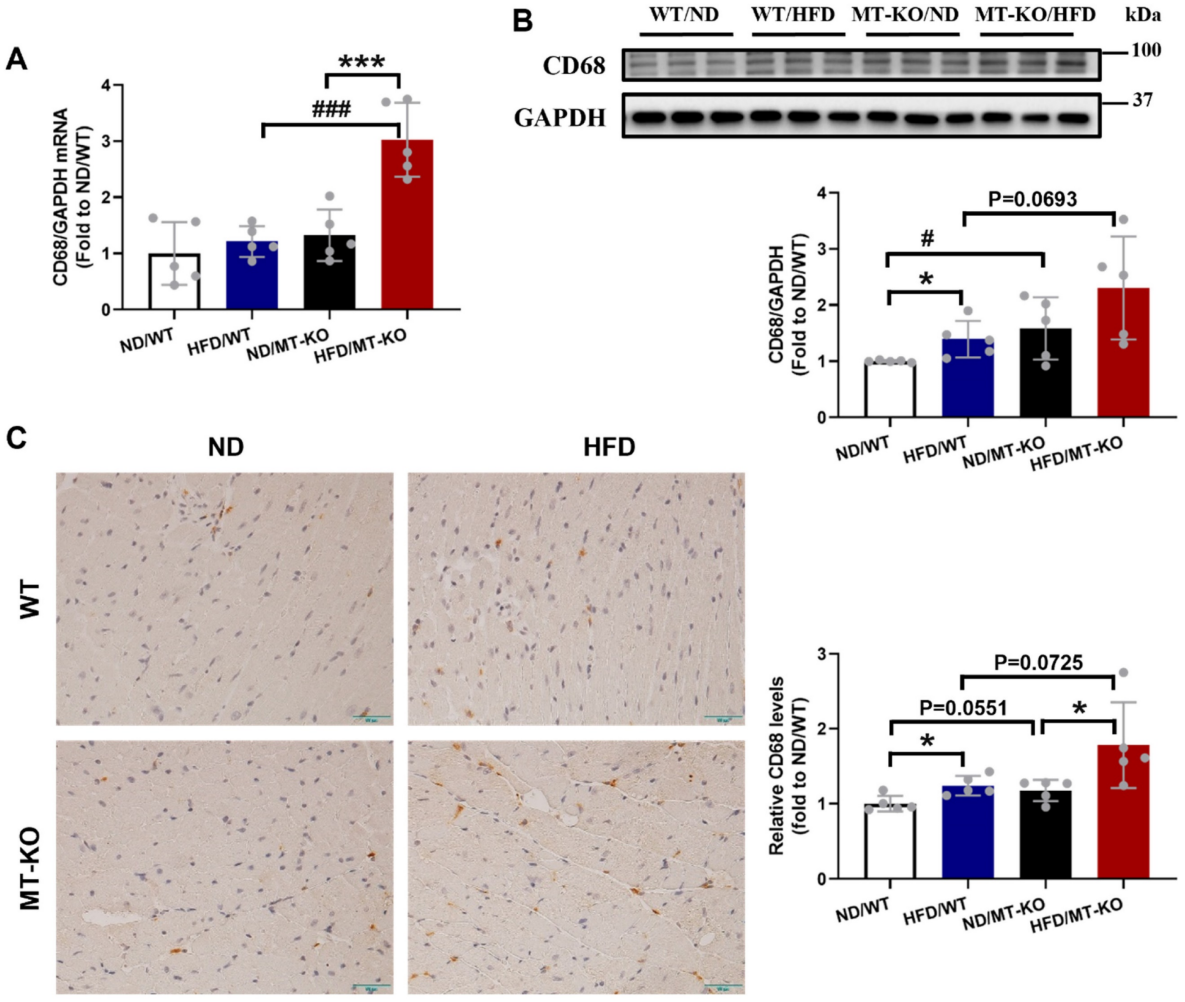
** The effect of *MT* knockout on HFD-induced macrophage infiltration into the heart.** A, mRNA expression of CD68 detected by qRT-PCR.B, Cardiac protein expression of CD68 evaluated by Western blot. C, Immunohistochemistry staining of CD68 results in the heart and quantitative analysis of IHC staining (brown considered positive staining; scale bar = 50 μm). Data are presented as Mean ± SD (n=5). *P<0.05, ***P<0.001, HFD vs. ND in WT and *MT*-KO groups, ^#^P<0.05,^ ###^P<0.001, *MT*-KO vs. WT in ND and HFD groups.

Obesity cardiomyopathy, characterized by macrophage infiltration in both adipose and cardiac tissues, is associated with low-grade inflammation 5,13. Therefore, we assessed mRNA and protein expression of CD68, a macrophage marker, in the heart following the 18-week HFD. Compared to the ND/WT group, HFD/WT and ND/*MT-*KO mice only trended increases in the transcriptional expression of CD68 (Fig. 4A) but significantly increased protein expression of CD68 (Fig. 4B). In contrast, HFD/*MT*-KO mice showed a synergistic increase in CD68 mRNA expression, whereas its protein expression did not significantly increase (Fig. 4A, B). Finally, IHC staining revealed similar mRNA and protein expression profiles for CD68 (Fig. 4C).

**Figure 5:**
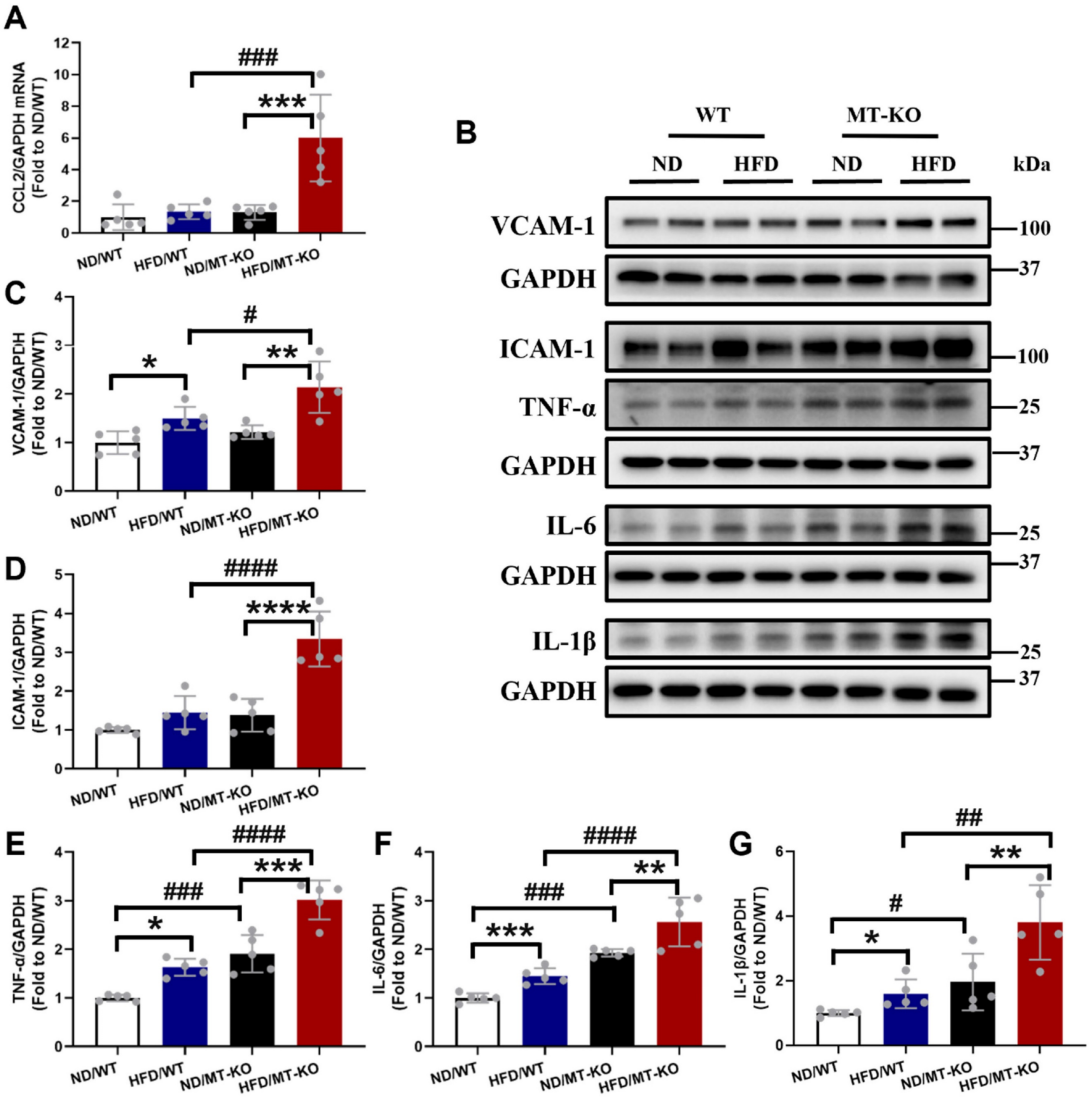
** The effect of *MT* knockout on HFD-induced secretion of adhesion molecules, proinflammation cytokines, and chemokines.** A, mRNA expression of chemokines including CCL2 evaluated by qRCT-PCR. B-G, Cardiac protein expression of VCAM-1(C), ICAM-1(D), TNF-α(E), IL-6(F), IL-1β(G) detected by Western blot. Data are presented as Mean ± SD (n=5). *P<0.05, **P<0.01, ***P<0.001, ****P<0.0001, HFD vs. ND in WT and *MT*-KO groups, ^#^P<0.05,^ ##^P<0.01,^ ###^P<0.001,^ ####^P<0.0001, *MT*-KO vs. WT in ND and HFD groups.

To elucidate the mechanisms by which macrophages infiltrate the cardiac tissues of *MT-*KO obese group, we evaluated the levels of macrophage-associated chemokine CCL2, along with ICAM-1 and VCAM-1. CCL2 signaling modulates macrophage recruitment and polarization. Additionally, ICAM-1 and VCAM-1 facilitate monocyte adherence to endothelial cells by regulating macrophage cellular chemotaxis and adhesion during inflammation (21-23). The qRT-PCR analysis for CCL2 mRNA showed that the combination of HFD and *MT*-KO significantly upregulated CCL2 transcription whereas either treatment alone did not (Fig. 5A). We then used western blotting to measure VCAM-1 and ICAM-1 protein expression. HFD or *MT* gene KO alone slightly or non-significantly upregulated VCAM-1 (Fig. 5B, C) and ICAM-1 expression (Fig. 5B, D). However, their combination substantially increased the expression levels of both adhesion molecules.

We then measured the expression of cardiac TNF-α, IL-6, and IL-1β. As shown in Fig. 5B, E-G, HFD and *MT* gene KO significantly upregulated these three inflammatory cytokines expression compared with the cytokine levels observed in WT mice receiving a ND. Moreover, the HFD-fed *MT-*KO group had higher cardiac TNF-α, IL-6, and IL-1β expression than its two corresponding control groups. Thus, both obesity and *MT*-KO increased cardiac macrophage infiltration and proinflammatory cytokine production, and the combination of *MT-*KO and obesity synergistically enhanced these responses.

#### The exacerbation of HFD-induced cardiac macrophage infiltration and inflammation by *MT* knockout may be associated with CARD9 activation-mediated signalling pathway

**Figure 6:**
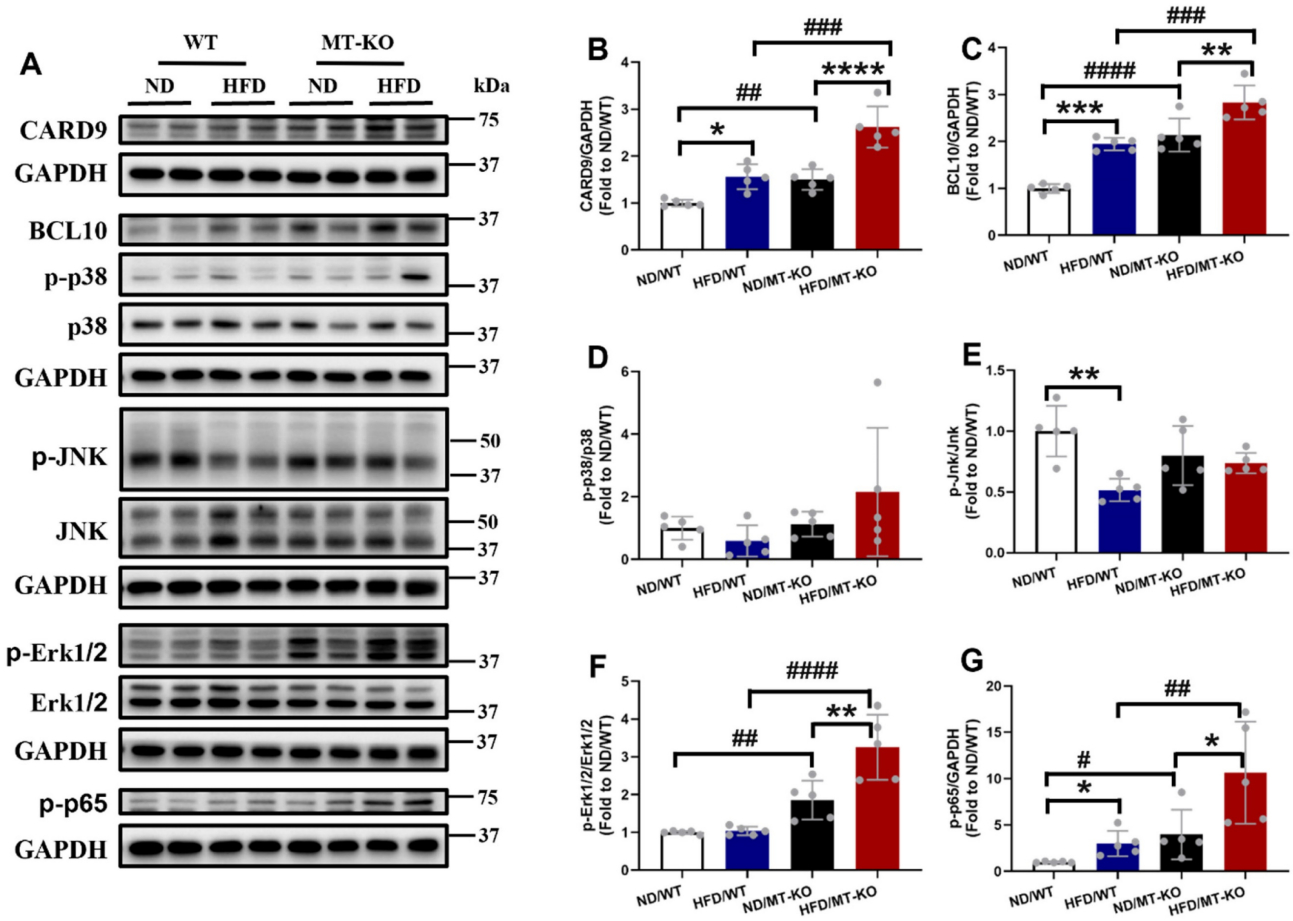
** The effect of *MT* knockout on HFD-induced activation of inflammation pathway.** A-G, Cardiac protein expression of CARD9 (B), BCL10 (C), phosphorylation and total p38 MAPK (D), phosphorylation and total JNK (E), phosphorylation and total ERK1/2 (F), and phospho-NF-κB p65 (G) were detected by Western blot, with GAPDH used as the loading control. Data are presented as Mean ± SD (n=5). *P<0.05, **P<0.01, ***P<0.001, ****P<0.0001, HFD vs. ND in WT and *MT*-KO groups, ^#^P<0.05,^ ##^P<0.01,^ ###^P<0.001,^ ####^P<0.0001, *MT*-KO vs. WT in ND and HFD groups.

In the myeloid lineage, including macrophages, dendritic cells, and neutrophils, CARD9 forms a CBM complex with BCL10 during innate immune responses, activates NF-κB and/or MAPKs, and stimulates proinflammatory cytokine production 7. Our previous *in vitro* study indicated that *MT* deletion excessively upregulated BCL10 expression and activated p38 MAPK in palmitate-treated cardiomyocytes compared to those in untreated WT controls 19. Therefore, we hypothesized that hyperactivation of the CARD9 signaling pathway in response to *MT* gene knockout would contribute to adhesion molecule and proinflammatory cytokine overproduction in *MT-*KO obese mice. Our results indicated that the CARD9 and BCL10 expression (Fig. 6A, B, C), in parallel to the phosphorylation level of NF-κB (Fig. 6A, G), but not those of p38 MAPK (Fig. 6A, D), JNK (Fig. 6A, E), or Erk1/2 (Fig. 6A, F), were higher in the HFD/WT and ND/*MT-*KO groups than in the ND/WT group. The extent of these increments was markedly greater in the HFD/*MT-*KO group.

**Figure 7:**
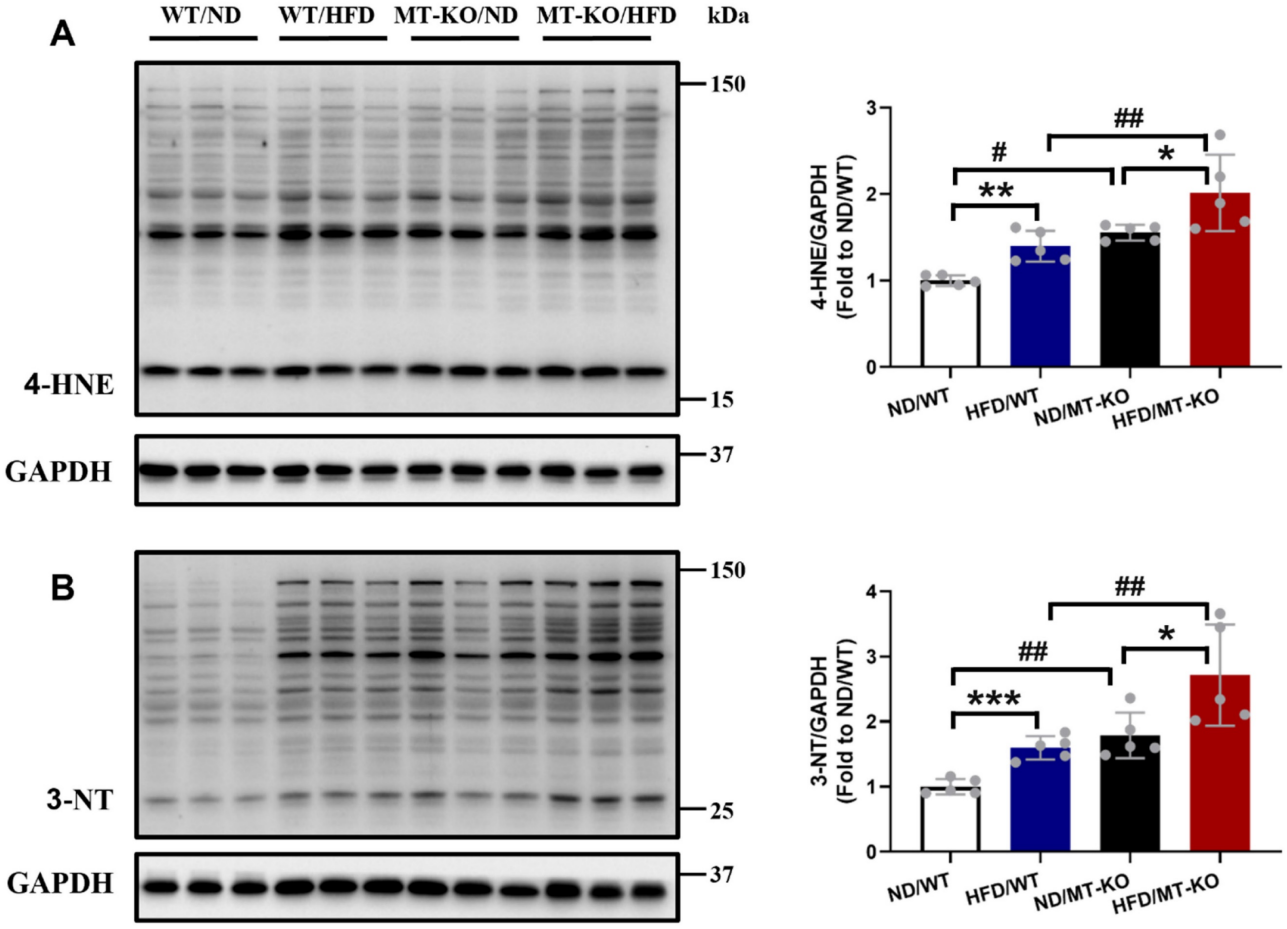
** The effect of *MT* knockout on HFD-induced oxidative stress damage in hearts.** Cardiac expression of 4-HNE (A) and 3-NT (B), as markers of oxidative stress damage, was detected by Western blot, with GAPDH used as loading control. Data are presented as Mean ± SD (n=5). *P<0.05, **P<0.01, ***P<0.001, HFD vs. ND in WT and *MT*-KO groups, ^#^P<0.05,^ ##^P<0.01, *MT*-KO vs. WT in ND and HFD groups.

To explore the reason for excessive inflammation, we examined cardiac oxidative stress damage by measuring the lipid peroxidation marker 4-HNE (Fig. 7A) and the protein oxidation marker 3-NT (Fig. 7B) using immunoblotting. The HFD-fed WT group exhibited upregulated levels of the two molecules compared to the ND-fed WT group (Fig. 7A, B). *MT*-KO mice with ND also had significantly increased expression of these two molecules in contrast with the ND-fed WT group (Fig. 7A, B). Meanwhile, upregulated levels of the two molecules were observed in the *MT*-KO group subjected to HFD, surpassing those in its two corresponding control groups (Fig. 7A, B). Thus, *MT*-KO aggravated obesity-induced oxidative stress and damage in the heart.

#### The exacerbation of HFD-induced cardiac macrophage infiltration and inflammation by *MT* knockout may be associated with trace element dyshomeostasis caused by *MT* knockout

**Figure 8:**
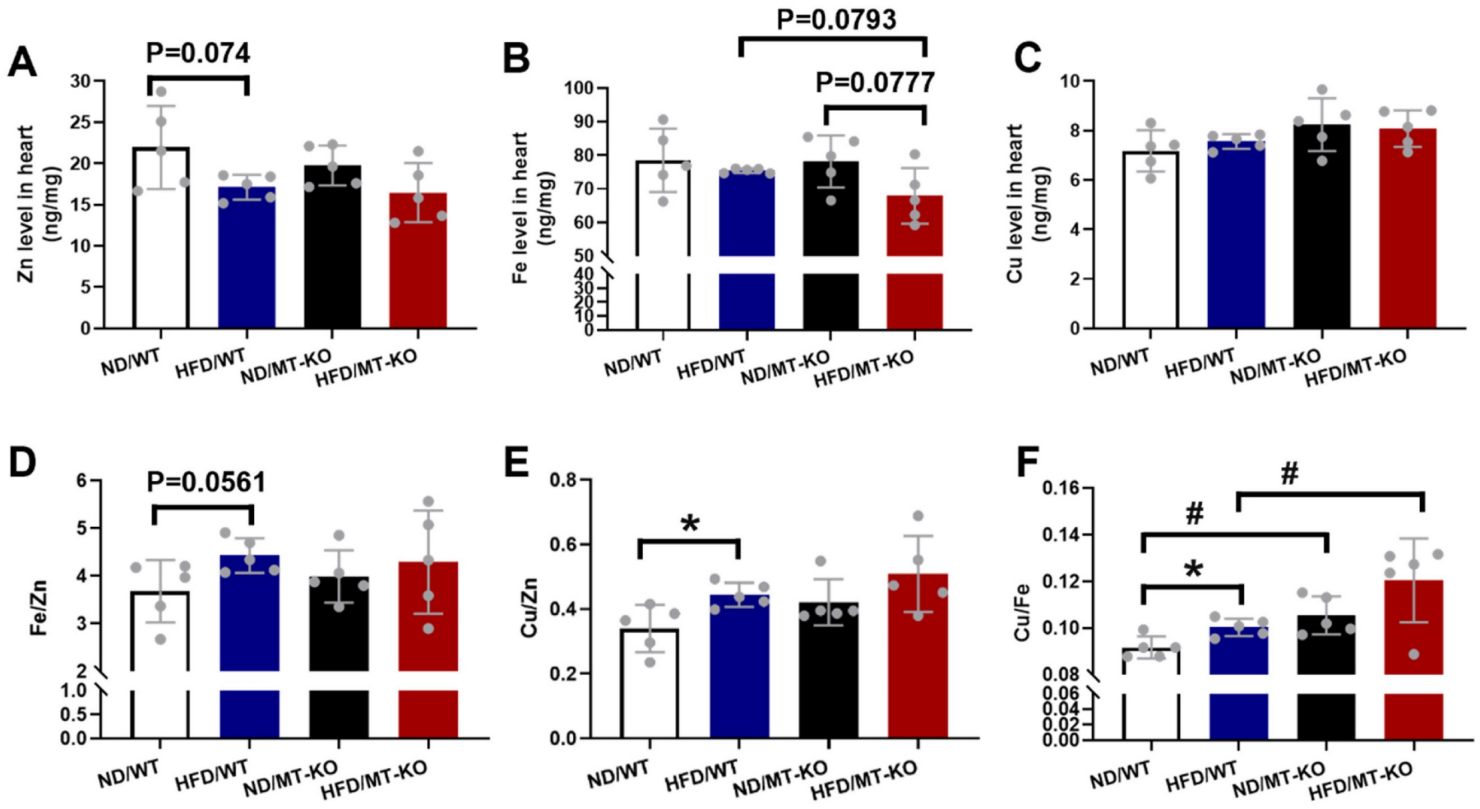
** The effect of *MT* knockout on HFD-induced changes of the essential metal level in the heart.** Zinc (Zn) (A), Iron (Fe) (B), Copper (Cu) (C) levels in the heart were measured in WT and *MT*-KO mice. The ratios of Fe/Zn (D), Cu/Zn (E), and Cu/Fe (F) in the heart were then calculated. Data are presented as Mean ± SD (n=5). *P<0.05, HFD vs. ND, ^#^P<0.05, *MT*-KO vs. WT in ND and HFD groups.

Trace element dyshomeostasis is involved in the inflammatory and oxidative stress damage in certain diseases 24. We measured Zn (Fig. 8A), Fe (Fig. 8B), and Cu (Fig. 8C) levels in the mouse hearts to assess how obesity and *MT*-KO affect trace element homeostasis. Obesity slightly reduced Zn levels in both WT and *MT-*KO groups compared to their corresponding ND controls (p > 0.05). However, no significant variations were detected between two groups receiving HFD. Fe levels were slightly decreased in *MT*-KO mice with HFD in contrast to HFD/WT and ND/*MT*-KO mice. Conversely, Cu levels were slightly elevated in ND/*MT*-KO and HFD/*MT*-KO groups compared to their WT controls, respectively (p > 0.05).

We then calculated Fe/Zn (Fig. 8D), Cu/Zn (Fig. 8E), and Cu/Fe (Fig. 8F). The HFD-fed WT group exhibited a minor increase in Fe/Zn compared to the ND-fed WT group. A HFD increased Cu/Zn and Cu/Fe in the WT group, whereas the *MT*-KO obesity mice presented with higher Cu/Fe than the HFD-fed WT group. These results suggest that *MT*-KO exacerbated obesity-mediated trace element dysregulation.

## Discussion

**Figure 9:**
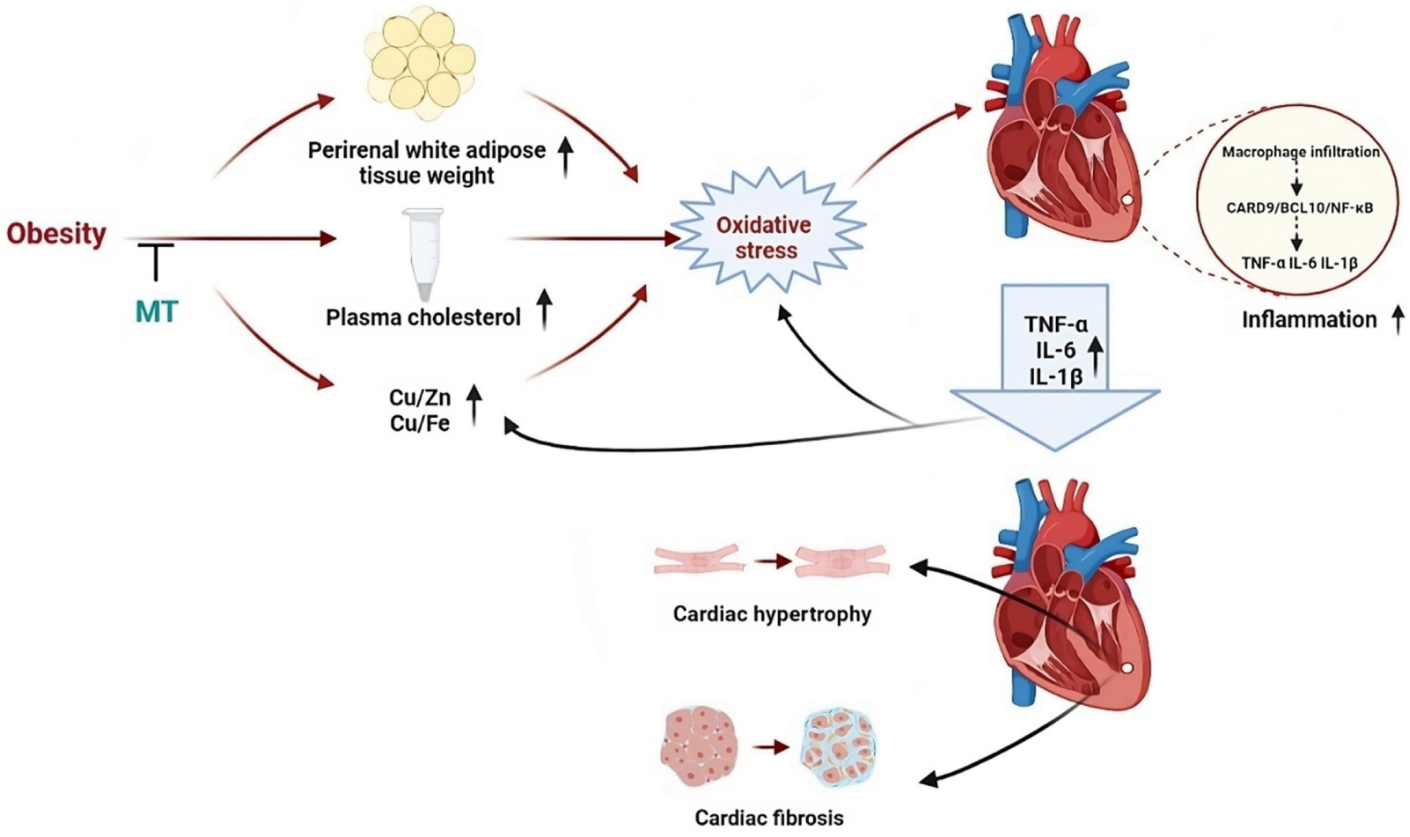
An illustration outlining the potential protective mechanisms of MT against obesity-induced adverse impacts on the heart that we studied.

In the present study, 18 weeks HFD feeding and *MT* gene knockout, respectively, caused obesity with glucose intolerance, hypercholesterolemia, essential trace element dyshomeostasis, oxidative stress, and inflammation without any significant changes in cardiac structure. The combination of obesity and *MT* deletion led to more severe chronic, low-grade inflammation and oxidative stress than either factor alone. In addition, this combination contributed to cardiac remodeling in the form of hypertrophy and fibrosis. These findings indicate that the endogenous stress protein MT protects the heart against HFD-induced oxidative stress and inflammation and consequent cardiac remodeling. Moreover, MT may prevent HFD-induced cardiac remodeling by inhibiting CARD9/BCL10-mediated NF-κB (but not MAPK) activation. Finally, HFD and/or *MT* gene knockout caused cardiac trace element dyshomeostasis which may trigger cardiac oxidative stress and activation of the CARD9 signaling pathway (Fig. 9).

An 18-week HFD (60% kcal fat) in WT mice led to obesity as shown by the augmentation of BW and perirenal white adipose tissue, as well as systemic metabolism disorder including impaired glucose tolerance and increased blood total cholesterol. These adverse metabolic changes induced by obesity were evident in previous studies 25,26. Moreover, *MT-*KO with ND feeding resulted in obesity accompanied by the lipid and glucose metabolism abnormalities as WT obese mice. This suggests that MT may help prevent obesity, as indicated by an earlier study 27. Here, the combination of HFD and *MT-*KO induced more severe obesity and higher blood cholesterol levels than either factor alone. This indicates that MT could also prevent HFD-induced obesity, as previously reported 28.

Eighteen weeks of HFD-induced obesity combined with *MT* deletion led to cardiac hypertrophy and fibrosis, whereas HFD-induced obesity only increased oxidative stress and inflammation but did not cause significant cardiac remodeling in the WT group. Thus, the basal MT level may be crucial for alleviating obesity-induced adverse cardiac effects, consistent with our previous *in vitro* study 19. Currently, there have been no prior *in vivo* studies exploring the protective effects of MT on HFD-induced obesity cardiomyopathy in *MT*-KO mice. However, we previously showed that MT alleviated diabetic cardiomyopathy and nephropathy 18,29. Here, obesity or *MT-*KO alone did not cause significant cardiac remodeling or dysfunction. Similarly, a previous study also did not find *MT-*KO showing cardiac remodeling or dysfunction even in the long term 18. However, three months of HFD feeding contributed to cardiac hypertrophy in our previous study 10. This finding differs from the results of the present study. This discrepancy may be attributed to variations in mouse strains and the ages at which a HFD was initiated across studies. In our previous study, we used the obesity-prone C57BL/6J strain 10, whereas we used the obesity-resistant 129S1 strain in the present study 30,31. Furthermore, HFD was initiated at 4 weeks of age in the previous investigation 10 but at 8 weeks in the present study.

Obesity-induced systemic metabolic disturbances includes impaired lipid and glucose metabolism and contributes to chronic low-grade cardiac inflammation 3. IL-6, TNF-α, IL-1β, and TGF-β, as downstream proinflammatory cytokines of NF-κB, produced and secreted by immune cells infiltrating the heart muscle, can contribute to cardiac remodeling 6,32. In addition, the activation of NF-κB can lead to the upregulated expression of chemokines and adhesion molecules, which contribute to immune cells infiltration in the heart 33. The inflammatory changes observed herein suggest that obesity and *MT-*KO synergistically affected macrophage infiltration in the heart, proinflammatory cytokine production, CARD9 signaling pathway activation, and, by extension, cardiac hypertrophy and fibrosis.

More specifically, in this process, we first observed macrophages infiltration, along with the expression of CCL2 and ICAM-1, VCAM-1, which are essential in recruiting macrophages and mediating macrophages adhesion to endothelial cells, showed an increasing trend in obese WT mice and *MT*-KO mice with ND, and a significant increase in *MT*-KO obese mice. Meanwhile, the expression of IL-6, TNF-α, IL-1β, and TGF-β had the same changes in parallel with macrophage infiltration. Consistent with previous studies, HFD-induced obesity caused macrophage infiltration into the heart and increased secretion of these proinflammatory cytokines 10,13, which contributed to cardiac remodeling. Moreover, diabetic *MT*-KO mice showed increased cardiac remodeling, mRNA levels of CCL2, and expression of TGF-β and IL-6 compared with diabetic WT mice 18. These findings indicate that MT prevented cardiac macrophage infiltration by inhibiting chemokine and adhesion molecule expression. Furthermore, the resulting limited inflammation could further restrain the progress of cardiac remodeling.

In addition, we discovered that CARD9 and BCL10 expression and NF-κB activation were markedly elevated in *MT*-KO obese mice than in WT obese or ND-fed *MT-*KO mice. However, MAPK (p38 MAPK, JNK, and Erk1/2) activation was not in parallel with the upregulated expression of CARD9 or BCL10. Our previous study reported that HFD-induced obesity led to cardiac remodeling, increased proinflammatory cytokine production, upregulation of CARD9 expression and phospho-p38 MAPK in the heart 19, whereas these obesity-induced adverse effects and p38 MAPK activation were blunted following *CARD9* knockout 13. Moreover, deletion of CARD9 and BCL10 decreased hypertension-induced cardiac fibrosis and electrical remodeling, and reduced NF-κB activity 34,35. These studies suggest CARD9 mediates its downstream inflammatory effect by triggering either MAPK or NF-κB signaling pathways under different conditions. Here our *in vivo* data suggest that CARD9/BCL10 may activate NF-κB, but not MAPKs, to mediate obesity- or MT-induced cardiac inflammation. Furthermore, CARD9/BCL10 may be implicated in the synergistic effects of *MT-*KO and obesity on cardiac remodeling.

Oxidative stress leads to protein modification, lipid peroxidation, and DNA damage 24, and is closely linked to inflammation in chronic disorders, including obesity, diabetes, atherosclerosis, and CVDs 36. Our earlier work showed that HFD-induced obesity significantly increases cardiac oxidative stress, which leads to cardiac hypertrophy 10. In line with these studies, our data indicate that obesity led to the downregulated expression of MT, along with increased protein oxidation (indexed by 3-NT) and lipid peroxidation (indexed by 4-HNE) in WT mice. Metallothionein decreases oxidative stress, thereby protecting the heart against metabolic disorders 18. MT prevents DCM, intermittent hypoxia-induced cardiomyopathy *in vivo*, and obesity-induced cardiac injury *in vitro* through its potent antioxidant effects against oxidative stress damage 18,19,37. In our study, *MT-*KO obese mice exhibited more severe oxidative stress-mediated heart damage than WT obese mice or *MT-*KO mice fed a ND. This finding suggests that the downregulation and deletion of MT contribute to decreased antioxidant capacity, leading to and exacerbating cardiac damage under obesity condition. Lipid peroxidation and reactive oxygen species (ROS) upregulate the expression of chemokines and adhesion molecules, thereby facilitating the recruitment of circulating inflammatory cells in the heart 38,39. Proinflammatory cytokines, in turn, increase ROS production-induced oxidative stress damage 40. Thus, our findings suggest that MT exerts its beneficial role in HFD by eliminating oxidative stress damage and further limits macrophage infiltration and proinflammatory cytokines-mediated cardiac remodeling.

Obesity causes essential trace elements metabolism disorders, including serum Zn deficiency 41, Fe deficiency 42, and Cu excess 43. The present study showed these trends in the myocardial concentrations of Zn, Fe, and Cu in WT obese mice in comparison to WT controls. As shown in our previous study, Zn deficiency increased oxidative stress and inflammation, which in turn contributed to cardiac remodeling and dysfunction 19. As a metal-binding protein, MT mostly binds Zn (and a little Cu) under physiological conditions, playing an important role in storing and donating Zn to other Zn-containing enzymes and transcriptional factors 44,45. Therefore, MT is crucial for maintaining metal homeostasis 45. However, the effects of MT on obesity cardiomyopathy and the cardiac essential metal remain largely understudied. We initially observed a decreasing trend in Zn levels in *MT-*KO obese mice compared to their counterparts on a ND, although this difference was not statistically significant. Moreover, cardiac Fe levels were slightly lower in *MT*-KO obese mice than in WT obese and ND-fed *MT-*KO mice.

Based on the existing antagonistic effects between Cu and Fe and between Cu and Zn 46, the application of essential trace element ratios (Fe/Zn, Cu/Zn, and Cu/Fe) has been appreciated as an index for the etiology, diagnosis, treatment, and prognosis of various diseases (47-50). Here, the Cu/Fe ratio was higher in the WT obese and ND-fed *MT-*KO mice than the WT control. Furthermore, this ratio synergistically increased in the *MT-*KO obese mice. In addition, Cu/Zn ratio exhibited similar trends to those of the Cu/Fe ratio. These findings are in concordance with the study by Wang et al., wherein the Fe/Cu and Zn/Cu ratios were lowest in the hair of the morbidly obese group compared to the slim, normal, and overweight or obese groups 46. Meanwhile, higher blood Cu concentrations, Cu/Zn ratio, and reduced Zn levels were found in patients with diabetes mellitus, or cardiomyopathy compared with normal controls 51,52. Abnormally high serum Cu/Fe ratios can distinguish individuals with progressive mild cognitive impairment (MCI) from cognitively stable MCI subjects 50. Moreover, the overproduction of free radicals/ROS induced by disruption of the essential metal homeostasis may lead to oxidative stress 24,53. Therefore, we hypothesize that simultaneous obesity and *MT* deletion triggered the metal metabolism disorders in the heart and led to the onset of oxidative stress damage and inflammation, which were responsible for the corresponding cardiac remodeling. In addition, the Cu/Fe and Cu/Zn ratios may provide markers for the stage of obesity-related cardiac metabolism in the heart and potentially facilitate the early intervention of MAO.

In conclusion, MT may prevent obesity-induced oxidative stress, inflammation, and, by extension, cardiac remodeling. Abnormal trace element metabolism may trigger oxidative stress, and MT could prevent this pathological process by inhibiting cardiac macrophage infiltration, downregulating CARD9 and BCL10, and activating NF-κB, but not MAPKs (Fig. 9). The discovery that MT-mediated CARD9 signaling activation is implicated in cardiac remodeling may serve as a foundation for future studies investigating the regulation of cardiac inflammation. This finding could also facilitate the identification of potential therapeutic targets for cardiac remodeling and dysfunction. Finally, the Cu/Fe and Cu/Zn ratios may enable clinical monitoring of the metabolic state of obesity.

## Figures and Tables

**Table 1:** Cardiac structure and function parameters measured by echocardiography.

	ND/WT	HFD/WT	ND/ *MT-*KO	HFD/ *MT-*KO
IVS;d (mm)	0.58±0.04	0.59±0.01	0.56±0.02	0.71±0.005**^#&^**
IVS;s (mm)	0.90±0.05	0.89±0.04	0.83±0.11	1.01±0.12**^&^**
LVID;d (mm)	3.84±0.11	3.6±0.09^*^	4.00±0.06^*^	3.93±0.05**^#^**
LVID;s (mm)	2.43±0.45	2.05±0.08	2.41±0.18	2.41±0.23
LVPW;d (mm)	0.73±0.04	0.73±0.02	0.72±0.03	0.84±0.01**^#&^**
LVPW;s (mm)	1.02±0.04	1.02±0.04	1.05±0.05	1.19±0.03**^#&^**
LV Vol;d (mm)	63.68±4.56	54.61±3.33^*^	69.93±2.50^*^	67.02±2.25**^#^**
LV Vol;s (mm)	22.05±11.32	13.66±1.41	20.48±3.47	20.65±5.08
EF (%)	66.38±13.93	75.03±1.66	70.84±4.24	69.36±6.59
FS (%)	37.01±9.38	43.02±1.45	39.86±3.70	38.74±5.08
LV mass (mg)	83.99±4.71	76.55±4.85	87.57±4.91	108.83±2.66**^#&^**

IVS: d: end-diastolic interventricular septum; IVS: s: end-systolic interventricular septum; LVID: d: left ventricular (LV) end-diastolic diameter; LVID: s: LV end-systolic diameter; LVPW: d: LV end-diastolic posterior wall; LVPW: s: LV end-systolic posterior wall; LV Vol: d: LV end-diastolic volume; LV Vol: s: LV end-systolic volume; EF: ejection fraction; FS: fractional shortening; LV mass: left ventricular mass. Data are presented as Mean ± SD (n=5). *P < 0.05 vs. ND/WT; **^#^**P<0.05 vs. HFD/WT: **^&^**P< 0.05 vs. ND/*MT*-KO.

## Abbreviations

MAO: metabolically abnormal obesity
CVDs: cardiovascular diseases
CAD: coronary artery disease
HF: heart failure
CARD9: caspase recruitment domain-containing protein 9
BCL10: B-cell lymphoma/leukemia 10
MALT1: mucosa-associated lymphoid tissue 1
CBM: CARD9/BCL10/MALT1
MT: metallothionein
Zn: zinc
Cu: copper
WT: wild type
KO: knockout
ND: normal diet
HFD: high-fat diet
BW: body weight
IPGTT: intraperitoneal glucose tolerance test
LV: left ventricular
LVID: LV internal dimension
LVPW: LV posterior wall thickness
IVS: interventricular septum
EF: ejection fraction
FS: fractional shortening
Fe: iron
ICP-MS: inductively coupled plasma mass spectrometry
WGA: wheat germ agglutinin
IHC: Immunohistochemistry
3-NT: 3-nitrotyrosine
4-HNE: 4-hydroxynonenal
VCAM-1: vascular cell adhesion molecule-1
ICAM-1: intercellular adhesion molecule-1
qRT-PCR: quantitative real-time polymerase chain reaction
COL1A1: collagen1A1
Fn: fibronectin
MCP-1/CCL2: monocyte chemoattractant protein-1
SD: standard deviation
HW: heart weight
ROS: reactive oxygen species
